# Diagnostic Performance of Diffusion MRI for differentiating Benign and Malignant Nonfatty Musculoskeletal Soft Tissue Tumors: A Systematic Review and Meta-analysis

**DOI:** 10.7150/jca.62131

**Published:** 2021-10-28

**Authors:** Qian Wang, Xinguang Xiao, Yanchang Liang, Hao Wen, Xiaopeng Wen, Meilan Gu, Cuiping Ren, Kunbin Li, Liangwen Yu, Liming Lu

**Affiliations:** 1 Department of Medical Imaging, Zhengzhou Central Hospital Affiliated to Zhengzhou University, 195 Tongbai Road, 455007, Zhengzhou, China.; 2 Clinical Research and Data Center, South China Research Center for Acupuncture and Moxibustion, Medical College of Acu-Moxi and Rehabilitation, Guangzhou University of Chinese Medicine, Guangzhou, China.; 3 Guangzhou University of Chinese Medicine, 510006, Guangzhou, China.; 4 Department of Neurology, Sun Yat-sen Memorial Hospital, Sun Yat-sen University, 107 Yanjiang West Road, Guangzhou, China.; 5 Department of neurological rehabilitation, Zhengzhou Central Hospital Affiliated to Zhengzhou University, 450000, Zhengzhou, China.; 6 Department of Medical Imaging, The First Affiliated Hospital of Zhengzhou University, Zhengzhou, China.

**Keywords:** diffusion weighted imaging, musculoskeletal, soft tissue tumors, meta-analysis

## Abstract

**Objective:** To evaluate the diagnostic performance of standard diffusion-weighted imaging (DWI), intravoxel incoherent motion (IVIM), and diffusion kurtosis imaging (DKI), for differentiating benign and malignant soft tissue tumors (STTs).

**Materials and methods:** A thorough search was carried out to identify suitable studies published up to September 2020. The quality of the studies involved was evaluated using Quality Assessment of Diagnostic Accuracy Studies-2 (QUADAS-2). The pooled sensitivity (SEN), specificity (SPE), and summary receiver operating characteristic (SROC) curve were calculated using bivariate mixed effects models. A subgroup analysis was also performed to explore the heterogeneity.

**Results:** Eighteen studies investigating 1319 patients with musculoskeletal STTs (malignant, *n*=623; benign, *n*=696) were enrolled. Thirteen standard DWI studies using the apparent diffusion coefficient (ADC) showed that the pooled SEN and SPE of ADC were 0.80 (95% CI: 0.77-0.82) and 0.63 (95% CI: 0.60-0.67), respectively. The area under the curve (AUC) calculated from the SROC curve was 0.806. The subgroup analysis indicated that the percentage of myxoid malignant tumors, magnet strength, study design, and ROI placement were significant factors affecting heterogeneity. Four IVIM studies showed that the AUCs calculated from the SROC curves of the parameters ADC and D were 0.859 and 0.874, respectively. The AUCs for the IVIM parameters pseudo diffusion coefficient (D*) and perfusion fraction (f) calculated from the SROC curve were 0.736 and 0.573, respectively. Two DKI studies showed that the AUCs of the DKI parameter mean kurtosis (MK) were 0.97 and 0.89, respectively.

**Conclusion:** The DWI-derived ADC value and the IVIM DWI-derived D value might be accurate tools for discriminating musculoskeletal STTs, especially for non-myxoid SSTs, using more than two b values, with maximal b value ranging from 600 to 800 s/mm^2^, additionally, a high-field strength (3.0 T) optimizes the diagnostic performance.

## Introduction

Soft tissue tumors (STTs) are a very heterogeneous group of tumors with various presentations, and still pose an important challenge in daily clinical practice 1, 2. Magnetic resonance imaging (MRI) is frequently used to characterize and grade soft tissue tumors. Some benign STTs, such as haemangioma and lipoma, have characteristic imaging features that can be correctly diagnosed with conventional MRI. However, for a large number of STTs with a nonspecific imaging appearance, conventional MRI is often not reliable for distinguishing malignant from benign soft tissue tumors 3-5.

In recent years, some studies have reported the usefulness of diffusion-weighted imaging (DWI) for benign and malignant STTs. DWI is a technique that is useful for qualitative and quantitative assessments of the motion of water in the tissue microenvironment 6-8. The apparent diffusion coefficient (ADC) is a numerical parameter that is calculated from DWI. According to some investigators, malignant STTs usually have lower ADC values due to increased tumor cell packing 9, 10, and the ADC values for myxoid tumors were significantly higher than those of non-myxoid tumors 6, 10. Other investigators reported overlapping ADC values for benign and malignant STTs 11. However, ADC can be influenced not only by molecular diffusion restriction but also by capillary perfusion effects, therefore, ADC might have a limitation in characterizing the lesion 12, 13. Currently, intravoxel incoherent motion (IVIM) DWI has proposed by Le Bihan 14, more accurately separates molecular diffusion and capillary perfusion effects in each voxel and therefore encompasses both the capillary perfusion-related parameter pseudo diffusion coefficient (D*), perfusion fraction (f), and the true molecular diffusion coefficient (D) 12, 13, 15. Although several studies have demonstrated the use of IVIM DWI for the differentiation benign and malignant tumors of pancreas, liver, and breast 16-19, little information is available regarding the usefulness of IVIM parameters for evaluation of musculoskeletal STTs.

Conventional DWI techniques always assume a Gaussian diffusion in which water molecules diffuse without any restriction. However, due to the complexity of complex biological tissues, the diffusion of water molecules tends to deviate from a Gaussian distribution, thereby limiting the effectiveness of conventional DWI. Diffusion kurtosis imaging (DKI) is an advanced non-Gaussian diffusion imaging technique that has been used to account for this deficiency and is more suitable for assessments of the tumor microenvironment 20-22. Previous studies have introduced the feasibility of DKI in brain, rectal, prostate, and breast tumors. However, few DKI studies have examined musculoskeletal STTs 20.

Previous studies were inconclusive because of insufficient samples, different histological tumor types and the use of different diagnostic algorithms, including b values, cut-off values, and MRI parameters 12, 13, 15, 23. We conducted a systematic review and meta-analysis to provide an overview of the diagnostic performance of standard DWI, IVIM DWI, and DKI, for differentiating benign and malignant musculoskeletal STTs.

## Methods

### Search strategy

We performed this meta-analysis according to the Preferred Reporting Items for Systematic Reviews-Diagnostic Test Accuracy (PRISMA-DTA) guidelines 24. A search of PubMed, CENTRAL (Cochrane Library), Embase, PMC**,** China National Knowledge Infrastructure (CNKI) and WANFANG databases from their inception until September 2020 was performed to find potentially qualified studies. Medical Subject Heading (MeSH) terms, keywords, or words appearing were used as the search strategy (S1). We did not impose language restrictions.

### Inclusion Criteria and Study Selection

#### Type of studies

The inclusion criteria were (1) cohort studies that included retrospective or prospective studies; (2) the sensitivity (SEN) and specificity (SPE) of DWI for distinguishing benign and malignant STTs were reported clearly in the study; and (3) sufficient information was available for calculating true-positive (TP), false-positive (FP), false-negative (FN), and true-negative (TN) values for the statistical analysis.

The exclusion criteria were (1) studies that were reviews, case reports or case series, abstracts, letters, comments, editorials, or animal studies and (2) studies that had insufficient data for reconstructing 2
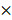
2 tables.

#### Type of patients

Patients with known musculoskeletal STTs who were referred for the initial lesion evaluation and had no history of treatment were included, all studies confirmed by histopathology, and excluded lipomatous tumors or vascular tumors.

#### Type of imaging technique

All studies used DWI, and ADC, DTI, DKI or IVIM parameters were calculated as the index test for differentiating benign and malignant STTs. The studies used other imaging techniques or used the DWI technique but did not pertain to the field of musculoskeletal STTs were excluded.

### Data Extraction

A data extraction spreadsheet was developed to collect all related information, then we summarized the information in table of baseline characteristics, imaging characteristics and diagnostic results.

#### Diagnostic results

The key information we extracted was the numbers of TP, FP, FN and TN using the DWI-derived parameter ADC; the IVIM DWI-derived parameters D, D*, and f; and the DKI-derived parameter mean kurtosis (MK) compared with histopathology. If TP, FP, TN, and FN were not reported, we calculated these values backwards using the indexes SEN, SPE, and number of malignant and benign lesions, and the results were rounded to the nearest whole number. For studies that reported multiple SEN and SPE due to the use of different combinations of b values, cut-off values, tumor pathologies, or different ROI placements, we extracted all the results. When studies included DWI and other MRI techniques, we only extracted the DWI data. When a study reported multiple histological classifications of STTs, we only extracted the data for benign and malignant tumors, as well as all soft tissue tumors (all STTs) and non-myxoid soft tissue tumors (non-myxoid STTs). When studies reported different ADC measurements, we only extracted the data for the minimal ADC value (ADC_min_) and mean ADC value (ADC_mean_). When studies had two readers, we extracted the reader with higher accuracy.

Other diagnostic results included the cut-off values, area under the curve (AUC), mean ADC, D, D*, f, and MK for benign and malignant STTs.

#### Basic study information

We extracted the following information: first author, date of publication, patient age, sex, number of patients, number of benign and malignant STTs, type of tumors (STTs or non-myxoid STTs), reference standard and type of study design (prospective or retrospective).

#### Basic Imaging information

We extracted the following information: (1) imaging parameters used for DWI (magnetic field strength, MRI machine and vendor, coil used, b values, slice thickness, sequence type, repetition time/echo time (TR/TE), matrix size, and field of view (FOV)); (2) ADC measurements (ADC_mean_ and ADC_min_); and (3) region of interest (ROI) placement (manually over the solid portion or other).

### Data Quality Assessment

Two reviewers independently assessed each study for quality and potential bias using the QUADAS-2 (Quality Assessment of Diagnostic Accuracy Studies, revised version) 25, which mainly consisted of 4 domains: (1) patient selection, (2) index test, (3) reference standard, and (4) flow and timing. When 5 or more than 5 terms were regarded as low risk, the study was classified as high quality. More than 70% of all included studies were high quality, and the quality of all included studies was regarded as high. Disagreements were reviewed in detail and subsequently settled by consensus.

### Statistical analysis

First, patient demographic characteristics and extracted covariates were summarized using standard descriptive statistics. Continuous variables are presented as the means and 95% confidence intervals (CIs), whereas categorical variables are presented as frequencies or percentages.

Second, the statistical analyses were performed using Meta-Disc 1.4 software (Ramón y Cajal Hospital, Madrid, Spain). Meta-analyses of standard DWI and IVIM DWI were performed as described below. First, heterogeneity between the included studies was evaluated. We evaluated the threshold effect by calculating the Spearman correlation coefficient between the logit of SEN and the logit of (1- SPE). The threshold effect was confirmed if the coefficient was >0.6 26. Apart from variations due to the threshold effect, heterogeneity might be generated from other related factors. Then, heterogeneity was determined using the Cochran Q test (*P*<0.05 indicating the presence of heterogeneity) and the *I^2^* test (with *I^2^*≤25% indicating low heterogeneity, 25%<*I^2^*≤50% indicating moderate heterogeneity, and *I^2^*>50% indicating significant heterogeneity) 26, 27. The SEN, and SPE of the included studies were pooled. According to the Cochrane review guidelines, if significant heterogeneity was present at *I^2^* > 50%, random-effects models were chosen; otherwise, fixed-effects models were used. We also calculated the AUC of the summary receiver operating characteristic curve (SROC). In general, a diagnostic tool is regarded as failed when AUC values range from 0.5 to 0.6, poor when AUC values range from 0.6 to 0.7, fair when AUC values range from 0.7 to 0.8, good when AUC values range from 0.8 to 0.9, and excellent when AUC values range from 0.9 to 1 26, 27.

Third, if significant heterogeneity was detected, we then performed meta-regression analyses, setting *P* < 0.05 to indicate a significant contribution to heterogeneity. Subgroups based on (1) ADC measurements (ADC_mean_ versus the ADC_min_); (2) the number of b values (=2 versus >2); (3) maximal b value (b_max_≤600 s/mm^2^ versus 600 s/mm^2^<b_max_ ≤800 s/mm^2^ versus b_max_>800 s/mm^2^); (4) magnet strength (3.0 T versus 1.5 T); (5) study design (prospective versus retrospective); (6) total number of patients (>60 versus ≤60); (7) ROI placement (manually over solid portion versus other); (8) tumor pathology (all soft tissue tumors versus non-myxoid soft tissue tumors); and (9) percentage of myxoid malignant tumors (>10% versus ≤10%) were analysed.

Last, due to the small sample size of the DKI studies, we did not perform a statistical analysis of the two studies and instead summarized their basic characteristics.

## Results

### Literature research

**Figure 1:**
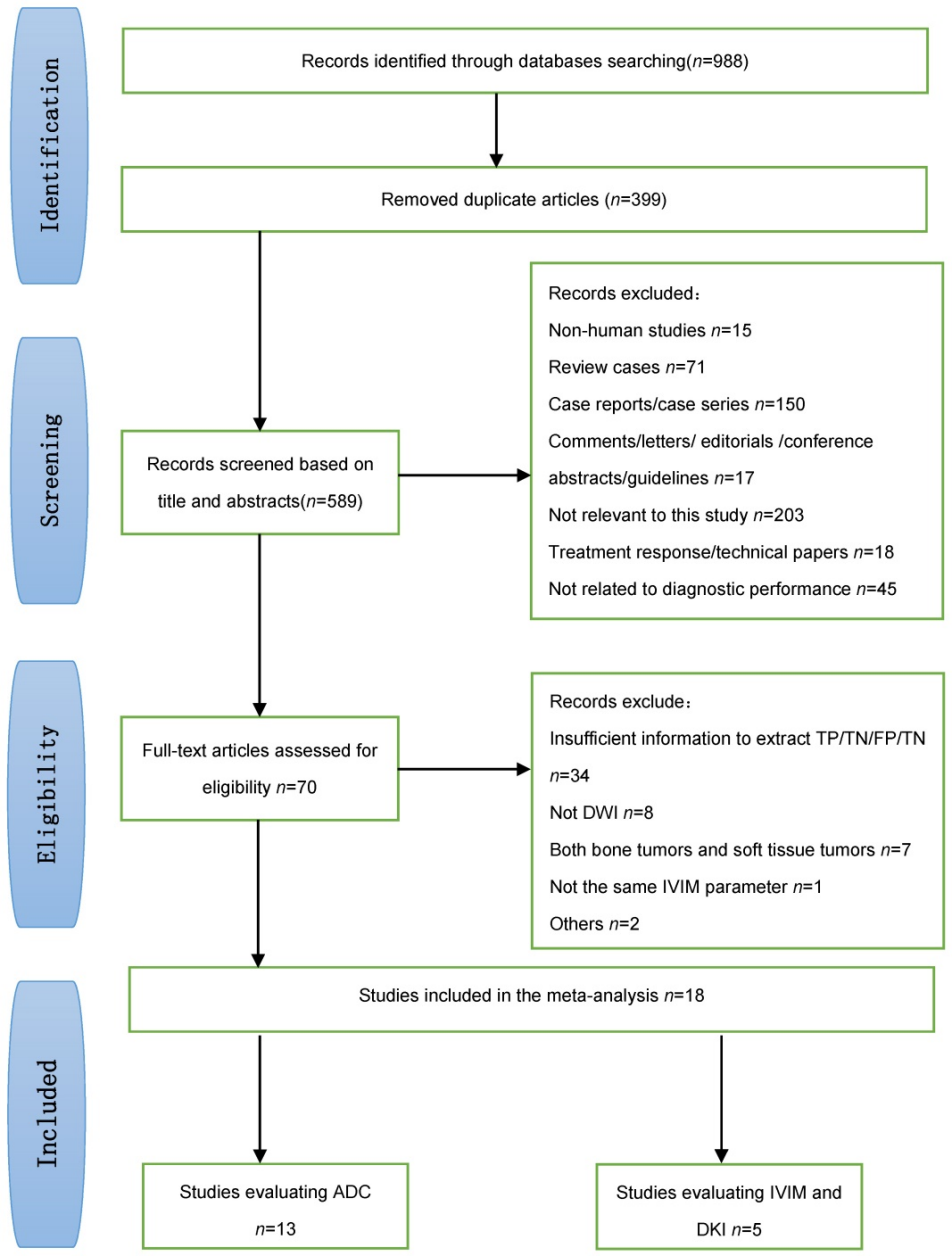
Flow diagram showing the study selection process for the meta-analysis.

**Figure 1** shows a flow diagram summarizing the literature search process. Ultimately, 18 studies 9, 15, 20, 23, 28-41 that met the eligibility criteria were included in our meta-analysis (13 studies evaluated standard DWI, 3 studies evaluated IVIM DWI, 1 study evaluated both IVIM and DKI, and 1 study evaluated DKI).

### Study characteristics

#### Patient characteristic

Ultimately, 18 studies examining 1319 patients with musculoskeletal STTs (malignant, *n*=623; benign, *n*=696) were included in this meta-analysis, which excluded lipomatous tumors and vascular tumors. **Table 1** provides an overview of the patient characteristics in the 18 eligible studies. We obtained 27 diagnostic results of standard DWI, 6 (22.2%) diagnostic results of IVIM DWI, and 2 (7.4%) diagnostic results of DKI for malignant and benign STTs.

#### Imaging characteristic

**Table 2** provides an overview of the MRI characteristics reported in the 18 eligible studies. The MR examinations were performed on a 1.5 T scanner in 8 (44.4%) studies and a 3.0 T scanner in 10 (55.6%) studies, which evaluated the diagnostic performance of DWI with b values ranging from 2 to 10. ADC maps were generated from DWI in the maximal b-value ranging from 300 s/mm^2^ to 2100 s/mm^2^. Seven (38.9%) studies used ADC_min_ as the differentiation criterion with a cut-off value ranging from 0.8-1.9, and 11 (61.1%) studies used ADC_mean_ as the differentiation criterion with a cut-off value ranging from 1.09-1.6. Three (16.7%) studies with IVIM DWI used all four IVIM parameters, including ADC, D, D*, and f, and 1 (5.56%) study only used two IVIM parameters, including ADC and D. Two (11.1%) studies reporting DKI used MK. In addition, 3 (16.7%) studies evaluated the diagnostic performance of ADC combined with conventional MRI.

### Quality of included studies

**Figure 2:**
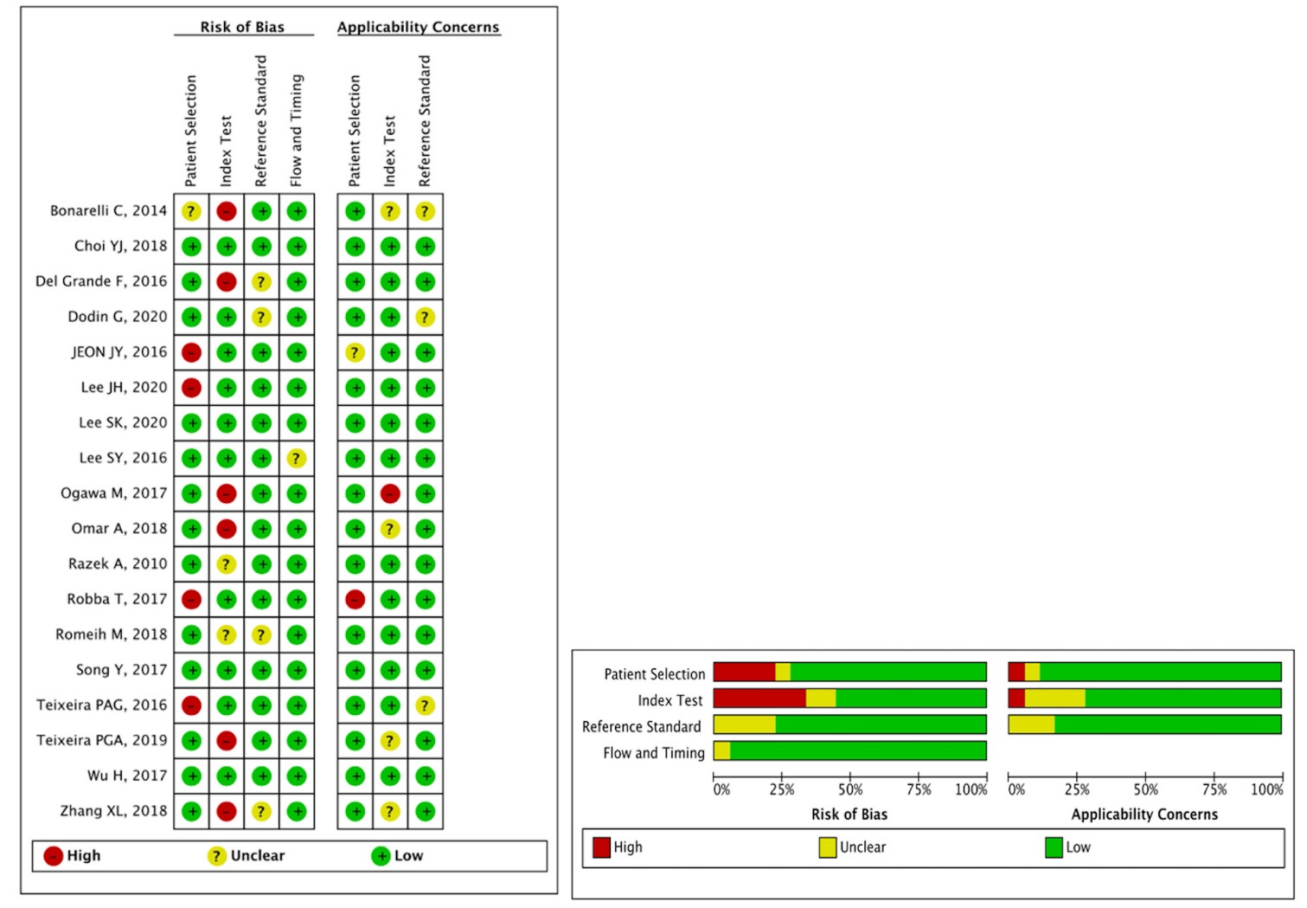
Summary of the risk of bias and applicability concerns across the included studies as assessed with QUADAS-2 forms.

Sixteen (88.9%) of the 18 included studies with 5 or more than 5 terms were regarded as low risk, and they were finally regarded as high quality; as a result, the quality of all studies was high. The detailed results for the QUADAS-2 scores for every study can be found in **Figure 2**.

In the patient selection domain, 1 (5.6%) study did not clearly describe the methods for patient selection, and 4 (22.2%) studies did not clearly report inappropriate exclusion. In the index test domain, 6 (33.3%) studies did not clearly report the threshold used, and 2 (11.1%) studies did not clearly report the blinding of the reference standard from MRI results. For the reference test domain, 4 (22.2%) studies were unclear, as no information was provided on whether the radiologists were blinded to the reference standard. For the flow and timing domain, 1 (5.6%) study was unclear because the time interval between MRI and the reference standard was not reported.

### Diagnostic Performance of Quantitative Assessment of standard DWI

**Figure 3:**
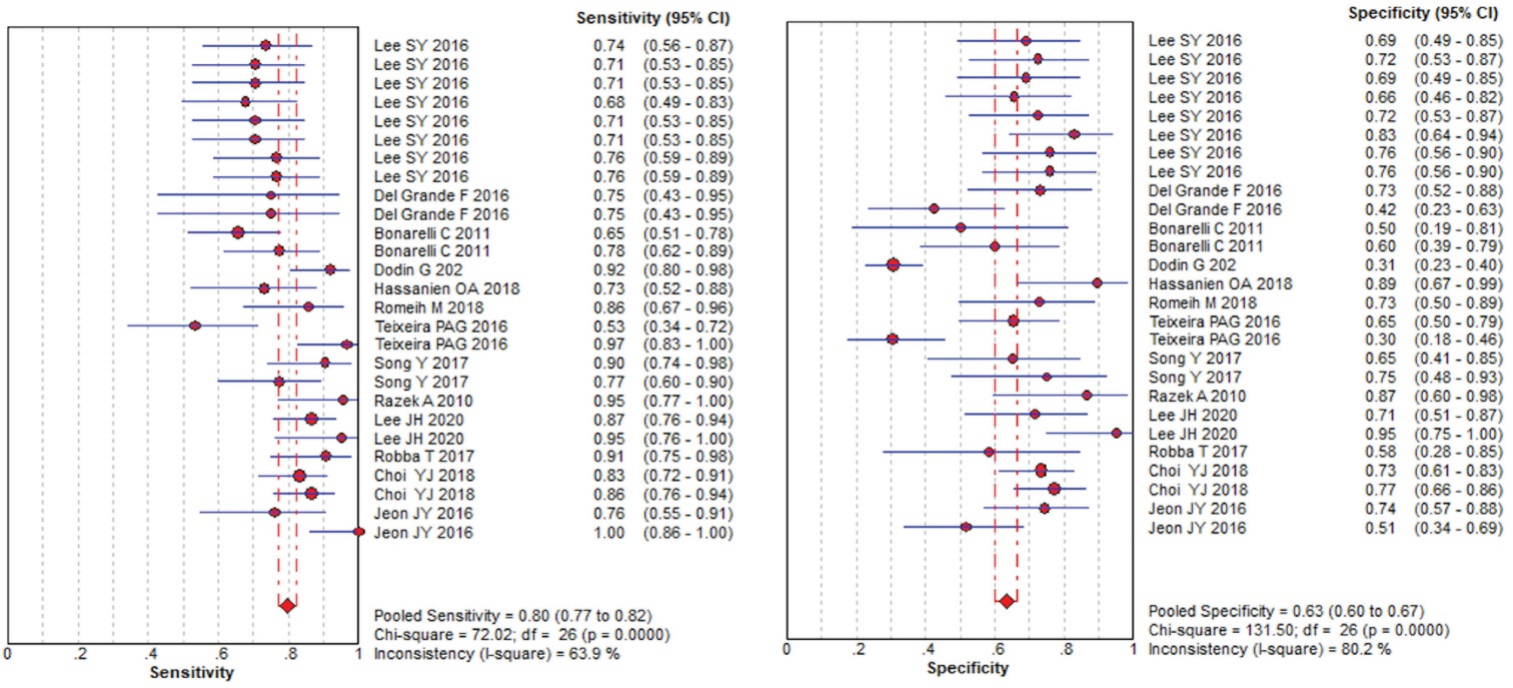
Pooled estimates of diagnostic performance of ADC values derived from DWI to differentiate benign and malignant STTs.

**Figure 4:**
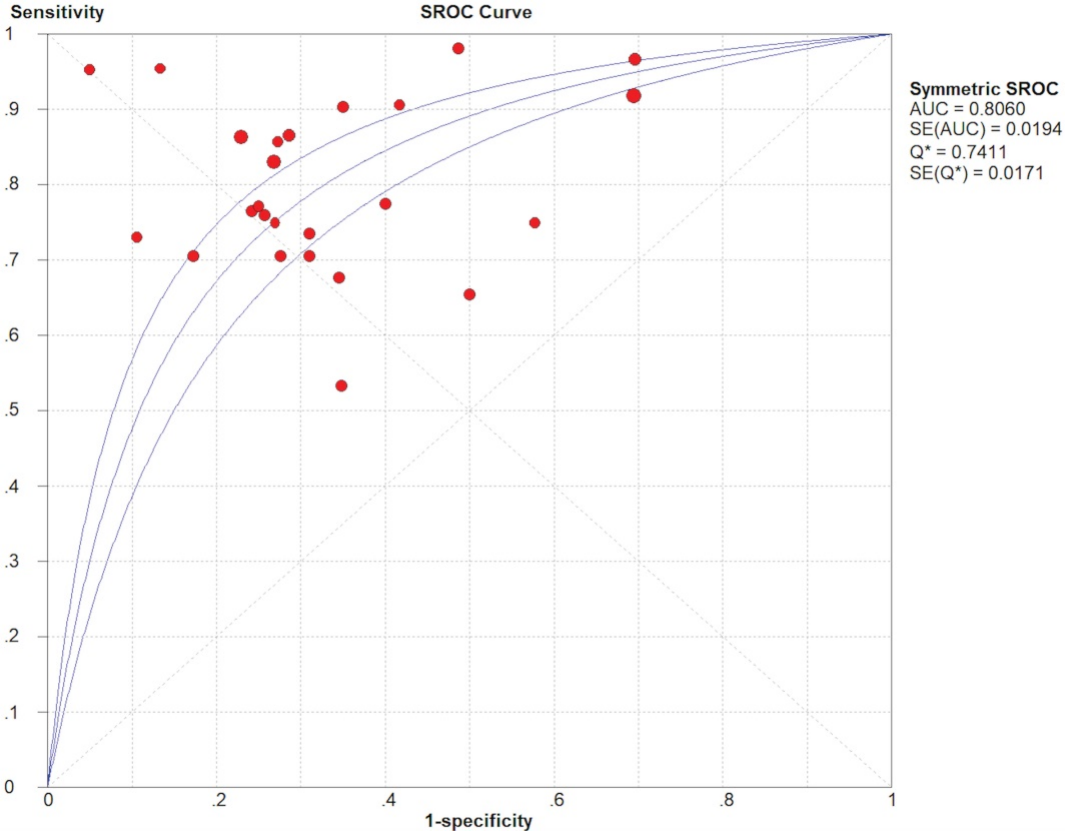
Summary receiver operating characteristic (SROC) curve of diagnostic performance of ADC values derived from DWI to differentiate benign and malignant STTs.

The diagnostic results of standard DWI for benign and malignant STTs are shown in** Table 3**. A large range of mean ADC values for malignant (0.74-1.58
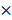
10^-3^ mm^2^/s), and benign lesions (0.97-1.92
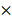
10^-3^ mm^2^/s) was reported. The SEN and SPE of the included diagnostic results ranged from 0.53 to 1.0 and from 0.30 to 0.95, respectively. The threshold effect was absent since the corresponding correlation coefficient was 0.047 between the logit of SEN and the logit of (1-SPE). The pooled SEN and SPE were 0.80 (95% CI: 0.77-0.82) and 0.63 (95% CI: 0.60-0.67), respectively. The forest plots are shown in **Figure 3**. The Q test indicated the presence of heterogeneity (Q=45.07; *p*=0.01), and the *I^2^* value of 42.3% revealed moderate heterogeneity. However, SEN (*I^2^*=63.9%) and SPE (*I^2^*=80.2%) indicated significant heterogeneity. The DOR was 8.61 (95% CI 6.93-10.72). The AUC was 0.806, and the SROC curve is shown in **Figure 4**. The results revealed the good diagnostic performance of ADC derived from DWI in differentiating benign and malignant STTs. When combined with conventional MRI in 3 studies 32, 33, 39, the AUCs increased to 0.97, 0.66, and 0.91, and the AUC of the SROC curve was 0.99, indicating perfect diagnostic performance.

#### Meta-regression and subgroup analyses

We next carried out meta-regression and subgroup analyses to explore the possible sources of heterogeneity, and the results of meta-regression and subgroup analyses are summarized in **Table 4**. Among the covariates that were considered a potential source of heterogeneity for sensitivity, the percentage of myxoid malignant tumors (*P*=0.041), magnet strength (*P* =0.019), study design (*P* =0.00), and ROI placement (*P* =0.018) were significant factors. Specifically, retrospective studies, studies that included myxoid malignant tumors less than 10%, studies that used a magnet strength of 3.0 T, and studies in which the ROI was not manually placed over the solid portion of the tumor reported higher SEN, SPE, DOR, and AUC values than the other subgroups.

The other factors, including the ADC measurements, number of b values, maximal b value, total number of patients, and pathology of the tumor, were insignificantly contributors to the heterogeneity. However, studies that only included non-myxoid tumors, studies that chose maximal b values ranging from 600 to 800 s/mm^2^, and studies that included more than 2 b values also reported slightly higher SEN, SPE, DOR, and AUC values.

### Diagnostic Performance of Quantitative Assessment of IVIM-DWI

**Figure 5:**
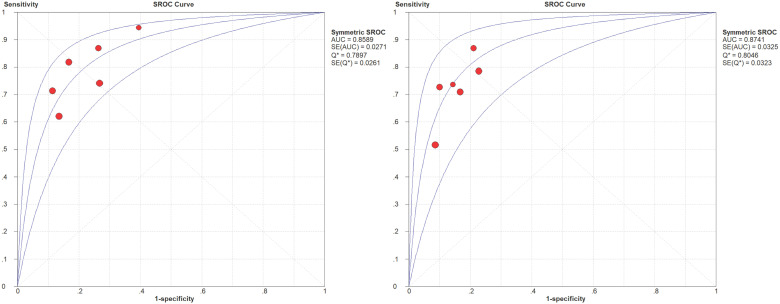
Summary receiver operating characteristic (SROC) curve of diagnostic performance of ADC and D values derived from IVIM-DWI to differentiate benign and malignant STTs.

**Table 5** shows the diagnostic results of IVIM DWI for benign and malignant STTs. A large range of mean ADC, D, D∗, and f values was reported. The threshold effect was observed for parameters ADC and D, since the correlation coefficients between the logit of SEN and the logit of (1-SPE) were 0.771 and 0.771, respectively, and the areas under the SROC curves (AUCs) were 0.859 and 0.874, respectively (shown in **Figure 5**). The Q test indicated the absence of heterogeneity (Q=2.07, *P*=0.839; Q=1.06, *P*=0.957; respectively) and the SEN (*I^2^*=47.5% and 41.8%, respectively) and SPE (*I^2^*=39.2%, 0%, respectively) exhibited moderate heterogeneity. The results revealed the good diagnostic performance of ADC and D derived from IVIM in differentiating benign and malignant STTs.

However, the threshold effect was absent for the parameters D* and f, since the correlation coefficients were -0.30 and 0.40, respectively. The Q test exhibited no significant heterogeneity (Q=7.29, *P*=0.12; Q=3.58, *P*=0.46; respectively). However, the pooled SEN was 0.68 (95% CI: 0.58-0.76) and 0.52 (95% CI: 0.43-0.61), the SPE was 0.68 (95% CI: 0.59-0.75) and 0.67 (95% CI: 0.59-0.74), and the AUCs were 0.736 and 0.573, respectively. The results were lower than other parameters and may indicate a failed-to-fair diagnostic performance in differentiating benign and malignant STTs.

### Diagnostic Performance of Quantitative Assessment of DKI

Two studies with DKI used the parameter MK. One study chose 4 values for the diffusion factor b (0, 1000, 1500, and 2000 s/mm^2^) in combination for diagnosis. The mean (±SD) MK values were 0.49 ± 0.17 and 1.14 ± 0.30 for benign and malignant tumors, respectively. At a cut-off of MK = 0.81, the SPE and SEN for the diagnosis of malignant tumors were 96.3 and 93.8%, respectively. The AUC was 0.97 for MK. Another study chose 5 values for the diffusion factor b (0, 100, 700, 1400 and 2000 s/mm^2^) in combination for diagnosis. The mean (±SD) MK values were 0.45 ± 0.97 and 0.82 ± 0.56 for benign and malignant tumors, respectively. At a cut-off of MK = 0.60, the SPE and SEN for the diagnosis of malignant tumors were 60 and 100%, respectively. The AUC was 0.89 for MK.

## Discussion

To our knowledge, our meta-analysis is the first to systematically compare all relevant standard DWI, IVIM DWI and advanced non-Gaussian diffusion techniques for DKI to evaluate the diagnostic performance in distinguishing benign and malignant musculoskeletal STTs. According to our search, few reviews pertain to musculoskeletal STTs. The aims of these reviews are divided into two types: one was to analyze DWI findings of different histological types of tumors and to compare the ADC values acquired, and the other was to review the various techniques of DWI acquisition and the utility of qualitative and quantitative methods of image interpretation, with an emphasis on optimal methods for ADC measurement 7, 42-45. There was only one meta-analysis evaluated the diagnostic potential of ADC values obtained from standard DWI in distinguishing malignant and benign STTs, which was estimated by the standard mean difference (SMD) 46.

The results of our meta-analysis revealed that standard DWI displays good diagnostic performance for discriminating between benign and malignant musculoskeletal STTs. However, a number of heterogeneities between studies of ADC need further consideration. By performing a meta-regression analysis, DWI performed at higher field strengths using 3.0 T MR systems was more effective than DWI performed using a 1.5 T system because the signal-to-noise ratio is influenced by the strength of the magnetic field, with higher fields producing a better signal-to-noise ratio and therefore a better quality of MRI for accurately drawing an ROI of the tumor 27, 46. The difference in the locations of ROIs also led to heterogeneity. Jeon, JY et al 39 reported that the AUCs of both ADC values extracted from the solid portion and whole tumor were nearly identical, but the ADC values extracted from the solid portion exhibited better diagnostic accuracy but lower sensitivity. The results of the subgroup analysis conducted in our study did not contradict this conclusion. Our study reported that setting the ROIs manually in the solid portion to extract ADC values had a lower AUC and sensitivity, which may make the ADCs unavailable to systematically clarify the characteristics and heterogeneity^26^, but this result was not reliable, because of the unstandardized technique, further studies will be needed to clarify the accuracy of this conclusion. The percentage of myxoid malignant STTs in every study might also be an important source of heterogeneity. Myxoid components of STTs are a potential source of inconsistency in the discrimination of malignant STTs from benign STTs, and significantly higher ADC values have been observed in myxoid-containing lesions 10, 30, 38, which may decrease the accuracy of ADC. Therefore, in our results, compared with the subgroup with a percentage of myxoid malignant tumors greater than 10%, the less than 10% subgroup showed a better diagnostic performance with a higher AUC and SEN values.

By other subgroup analysis, we found that maximal b value (600<b≤800), the number of b value (>2), pathology of tumor (non-myxoid tumor) and the ADC measurement were insignificantly contributed to the heterogeneity, but also have clinical diagnosis value, and probably affect test performance. The use of a higher b value for DWI scans has been suggested to result in ADC values with a lower effect of blood perfusion and a better response to the diffusion of water molecules within the tissue 27, and the use of more b values might achieve better separation. Although the use of at least three values ranging from 150-900 mm^2^/s was advocated in the Consensus Conference held in Toronto in 2008 47, the optimal number and scale of b value are still controversial 31, 32, and our results may provide reference values for clinical applications to some extent. In the subgroup analysis, the best results were obtained from the study group in which the tumor pathology was non-myxoid tumors, with an AUC of 0.94. As mentioned above, ADC values for the percentage of myxoid malignant tumors may be affected by the myxoid matrix within the tumor, which makes a radiological diagnosis based on ADC values alone quite difficult 6, 29. However, most myxoid tumors have suggestive characteristics on conventional MRI, appearing as “fluid-like” lesions with very high signal intensity on T2-weighted images, low signal intensity on T1-weighted images and variable enhancement after gadolinium injection 37, 48. Thus, the combination of these techniques in conventional MRI may lead to fewer misdiagnoses. As shown in our meta-analysis, 3 studies 32, 33, 39 analysed the diagnostic performance of DWI combined with conventional MRI, and the AUC of the SROC curve was 0.99, which was significantly higher than DWI alone and indicated perfect diagnostic performance. In clinical practice, for tumors with no typical myxoid characteristics according to conventional MRI, the ADC technique can be added to increase the diagnostic SEN. Moreover, no consensus has been reached regarding the most useful ADC measurements. A previous review 42 reported that the ADC_min_ value had a better diagnostic performance for discriminating STTs because it may provide more insights into the cellular composition, while the ADC_mean_ value may be diluted because of myxoid, cystic or fewer cellular regions included in the ROI. In contrast, our study revealed that the pooled SEN, SPE and AUC of both ADC_min_ and ADC_mean_ values were nearly identical, which may be important for radiologists when the ADC of DWI is applied in daily clinical practice.

Furthermore, our meta-analysis revealed that IVIM DWI-derived D and ADC values showed good diagnostic performance for differentiating malignant from benign STTs, and D would be slightly better than ADC because D eliminates the contributions of tissue perfusion to reflect tissue diffusivity more precisely than ADC^49^. Our results contradicted a previous study by Wu H and Lee SK et al 15, 40 showing that the AUCs of ADC were slightly better than those of D based on the ROC analysis. We also noted that the ADC and D values gradually decreased from benign to malignant tumors, in which water molecule diffusion was more impeded due to the increase in cell density and decrease in the extracellular space 15, 17, 50. However, IVIM-derived D* and f failed to show fair diagnostic performance. D* is considered proportional to the mean capillary segment length and average blood velocity and may chiefly reflect tumor vascularity 15, 51 Although our results showed a distinct increase in D* from benign to malignant soft tissue tumors, the diagnostic performance was fair. The f value may correlate with the amount of normal angiogenesis with intact vessels in terms of basement membrane thickness and pericyte coverage and may be an indicator of intact vascular permeability. Perhaps due to variability in histological grades or varying tumor vascularity among the tumor types 15, 52, the diagnostic performance of the f value was poor. Further studies with larger numbers of subjects are needed to confirm our findings.

According to previous studies, DKI parameters are sensitive to noise effects and the degradation of image quality that strongly influence nonbrain imaging, and the reliability may be insufficient, especially for nonbrain imaging 20, 53. Few DKI studies on musculoskeletal STTs have been conducted, and we only included 2 studies. They both revealed that the DKI-derived MK parameter showed good diagnostic performance for differentiating malignant from benign STTs, which was presumed to be correlated with the complexity of the tumor microenvironment 39. However, the result is inconclusive due to the small number of publications, and these studies did not include patients with myxoid sarcoma or chondrosarcoma.

## Limitations

Our study has some limitations that cannot be ignored. First, the overrepresentation of a sample may be a limitation of our pooled estimates because we included multiple sensitivities and specificities from the same authors investigating the same population. Second, our study did not include DTI studies. DTI is one of the conventional DWI techniques that evaluate the three-dimensional (3D) motion of protons in tissues, providing quantitative data on the amount and directionality of random movement of water molecules. DTI is a feasible technique that is mainly used to evaluate peripheral nerve tumors and soft tissue tumors arising around nerve structures 54, 55. Therefore, these studies did not conform to our study selection criteria. Last, although the possible sources of heterogeneity were explored, the different MRI vendors, sequences, imaging parameters, anatomical locations and tumor physiologies and their potential impacts on conclusions should not be neglected; thus, well-conducted investigations using a standardized methodology are needed to confirm the utility of DWI for discriminating between benign and malignant STTs.

## Conclusions

In conclusion, the DWI-derived ADC value and the IVIM DWI-derived D value might be an accurate tool for discriminating STTs, especially for non-myxoid SSTs; the use of more than two b values, a maximal b value between 600 and 800 s/mm^2^, and high field strength (3.0 T) may optimize the diagnostic performance. The use of DWI combined with conventional MRI might help to improve the diagnostic accuracy. However, more high-quality studies with larger samples are warranted to verify a standardized methodology in clinical practice.

## Figures and Tables

**Table 1:** Baseline characteristics of included studies

Authoryear	County	Study type	NO. Patients(M/F)	Age (years)mean ± SD (range)	NO. Tumors(M/B)	Pathology of tumor	Percentage of myxoid MTTs (%)	Reference standard	MRI Technical*	ROI placement	Diffusion parameters evaluated
Lee SY^ (32)^ 2016	Korea	Retro	63(35/28)	51(17-90)	34/29	All STTs	20.3(5/34)	HP	conventional MRIDWI	manually/solid tumor area	ADC_min_ADC_mean_
Del Grande F^ (36)^2016	USA	Pros	39(20/19)	malignant53.7±21.5(21-82)benign44.6±21.1(1-87)	12/27	All STTs	8.3(1/12)	HP+follow up	conventional MRIDWI static CE-MR dynamic CE-MR	manually/largest area of tumor	ADC_min_ADC_mean_
Bonarelli C ^(33)^2014	France.	Pros	65(35/ 30)	50(19-86)	24/41	All STTs	25(6/24)	HP	conventional MRIDWI	Manually/semi- automatic/Cystic solid, mixed	ADC_min_ ADC_mean_
Dodin G ^(34)^ 2020	France	Pros	288 (148/ 140)	50±17 (18-90)	104/184	non-myxoid STTs	0	HP+follow up	conventional MRIDWI/DCE/MRS	manually/solid tumor area	ADC_min_
Hassanien OA^ (35)^ 2018	Egypt	Pros	45(13/32)	42±18.5 (9-72)	21/24	All STTs	9.5(2/21)	HP+follow up	conventional MRIDWI	manually/fleshy tumor area	ADC_mean_
Romeih M ^(9)^2018	Egypt	Pros	50	33(1.5-75)	28/22	All STTs	14.3(4/28)	HP	conventional MRIDWI	NA	ADC_mean_
Teixeira PAG ^(37)^2016	France	Pros	76	NA	30/46	All STTs	30(9/30)	HP	conventional MRIDWI	manually/solid tumor area	ADC_min_ ADC_ratio_
Song Y ^(38)^2017	Korea	Retro	51	46.2(7-81)	31/20	non-myxoid STTs	0	HP	conventional MRIDWI	manually/solid tumor area	ADC_min_ADC_mean_
Razek A ^(28)^2010	Egypt	Retro	37(22/15)	41(4-68)	23/14	non-myxoid STTs	0	HP	conventional MRIDWI	manually/solid tumor area	ADC_mean_
Lee JH ^(29)^2020	Korea	Retro	95(49/46)	45.6(12-80)	29/66	All STTs/non-myxoid STTs	27.6(8/29)	HP+follow up	conventional MRIDWI	manually/solid tumor area	ADC_mean_
Robba T ^(30)^ 2017	Italy	Retro	46(27/19)	57(12-85)	34/10	All STTs	29.4(10/34)	HP+follow up	conventional MRIDWI	manually/solid tumor area	ADC_mean_
Choi YJ ^(31)^ 2018	Korea	Retro	136(68/68)	57.2(16-86)	63/73	All STTs	5.5(4/73)	HP	conventional MRIDWIDCE	manually/solid tumor area	ADC_min_ADC_mean_ADC_max_
Jeon JY ^(39)^2016	South Korea	Retro	60(30/30)	48.5(5-80)	35/25	All STTs	4.0(1/25)	HP	conventional MRIDWI	manually/solid + entire tumor area	ADC_mean_
Wu H ^(15)^ 2017	China	Pros	65(35/39)	48.4(12-75)	47/23	All STTs	8.7(2/23)	HP	conventional MRIIVIM-DWI	manually/solid tumor area	ADCstd, D, D* f
Teixeira PAG^(23)^ 2019	France.	Pros	64(23/41)	52±17(18-92)	35/29	All STTs/non-myxoid STTs	6.25(4/64)	HP	conventional MRIIVIM-DWI	manually/solid tumor area/entire area	ADCstd, D, D* f
Lee SK^ (40)^ 2020	Korea	Retro	67(30/37)	55±15(18-82)	35/32	All STTs/non-myxoid STTs	18.7(6/32)	HP	conventional MRIIVIM-DWIDCE	manually/solid tumor area	ADCstd, D, D* f
Zhang X^ (41)^2018	China	Pros	26	45.3±19.9(6-77)	11/15	non-myxoid STTs	0	HP	conventional MRIIVIM-DWIDKI	manually/solid tumor area	IVIM: ADCstd, D, D* f DKI:MK MD
Ogawa M^ (20)^ 2017	Japan	Pros	43(25/18)	62(18-90)	27/16	non-myxoid STTs	0	HP+follow up	conventional MRIDKI	manually/solid tumor area	MKADC_min_

*Studies included all MRI technique, but we only extracted the DWI part when extract data. MTTs: malignant soft tissue tumors; Retro: retrospective; Pros: prospective; All STTs: all soft tissue tumors; non-myxoid STTS: non-myxoid soft tissue tumors; ADC_min_: minimal ADC value; ADC_mean_: mean ADC value; ADCmax: maximal ADC value; ADCratio: dividing tumor minimal ADC by muscle ADC values; M/F: male/female; M/B: malignant/benign.

**Table 2:** Imaging parameters of included studies

Author, year	MRI vendor	Strength (T)	coil *	Sequence type	b-values (s/mm2)	TR/TE	Matrix size *	Slice thickness (mm) *	FOV (mm2) *
Lee SY^ (32)^ 2016	Verio; Siemens	3.0	phased-array /eight-channel extremity coil	SS-SE-EPI	0.300.800.1400	5000-8700/71-85	64×45-20×128	2-5	80-220
Del Grande F^ (36)^ 2016	Verio, Skyra, TrioSiemens	3.0	phased-array /eight-channel extremity coil	SS-EPI	50.400.800	4500-7600/80-84	NA	NA	NA
Bonarelli C^ (33)^ 2014	Signa HDxt, GE	1.5	dedicated coils	SS-PGSE-EPI	0.600	5000/minimal	128 × 80	*	*
Dodin G^ (34)^ 2020	Signa HDxt, GE	1.5	NA	SS-PGSE-EPI	0.600	4000-6225/66-88	128×80-256×256	5-6	190×260-484×280
Hassanien OA^ (35)^ 2018	Signa EXCITE GE	1.5	body phased-array coil	SS-EPI	0.500.800	8000-8500/50-60	128×64	4-10	25-30
Romeih M^ (9)^ 2018	Achieva XR Philips	1.5	surface coil	SS-SE-EPI	0.400.800	NA	128×64	4-10	NA
Teixeira PAG^ (37)^ 2016	Signa HDxt, GE	1.5	dedicated coils	SS-PGSE-EPI	0.600	5000/minimal	128×80	6	*
Song Y^ (38)^ 2017	Achieva TX; Philips	3.0	RF coils	SS-SE-EPI	0.400.800	NA/61-69	128×128-256×256	5	160-350
Razek A^ (28)^2010	Symphony, Siemens	1.5	NA	multislice SE-EPI	0.500.1000	10,000/108	256×128	5	250-300
Lee JH^ (29)^ 2020	Intera Achieva/ Ingenia Philips	3.0	RF coils	SS-SE-EPI	0.400.800	5,000/61-69	128×128-256×256	5	160-350
Robba T^ (30)^2017	Signa Excite HD, GE	1.5	NA	SS-EPI	0.800	NA	NA	NA	NA
Choi YJ^ (31)^2018	Tim Trio, Siemens	3.0	variable coils	SS-EPI	0.50.500.800.1400	4400-7100/56-88	100-430	2-7	100-400
Jeon JY^ (39)^2016	AchievaTM, Philips	3.0	dedicated surface coils	SS-EPI	0.400.800	5000-5200/61-85	70×70-128×128	2.5-5	100-250
Wu H^ (15)^ 2017	Achieva 1.5T, Philips	1.5	body or surface coil	SS-PGSE-EPI	0.10.20.30.40.50.75.100. 150.300.500.800	4500/65	128×136	5	380×380
Teixeira PAG^ (23)^ 2019	Discovery MR750, GE	3.0	dedicated coils	SE—EPI	20.40.60.80. 100.200.300. 500.700.900	4000-17000/<80	96×128	5	180×180-500×500
Lee SK^ (40)^ 2020	MAGNETOM Verio, Siemens	3.0	NA	SS-SE-EPI	0.25.50.75.100. 200.300.500.800	NA	NA	NA	NA
Zhang X^ (41)^2018	Skyra, Siemens	3.0	eighteen-channel extremity coil	GRAPPA	IVIM:0.10.20.30.40.50.75.100.150.200.400.800, 1000,1500DKI:0.100.700.1400.2100	IVIM:3000/61DKI:3370/68	120×120	4	IVIM:203 ×203DKI:200 ×200
Ogawa M^ (20)^2017	Hitachi	3.0	NA	SE-EPI	0.1000.1500.2000	3000/84	64×64	5	250×250

SS-EPI: single shot echo planar; SS-PGSE-EPI: single-shot pulsed gradient spin-echo echo planar; SS-SE-SEP: single shot, spin-echo echo planar; SE-EPI: spin-echo echo planar; multislice SE-EPI: multislice spin-echo echo planar; RF coils: radiofrequency coils.*Depending on mass size and location, adapted to the patient anatomy and tumor size.

**Table 3:** Diagnostic results of ADC for benign and malignant soft tissue tumors

Author, year	b-values (s/mm^2^)	ADC measurements	Cutoff values (×10^-3^mm^2^/s)	TP	FP	FN	TN	ADC of benign tumors (×10^-3^mm^2^/s)	ADC of Malignant tumors (×10^-3^mm^2^/s)
Lee SY, 2016	0.300	ADC_mean_	1.60	25	9	9	20	1.92±0.63	1.30±0.55
	0.800	ADC_mean_	1.30	24	8	10	21	1.60±0.53	1.09±0.49
	0.1400	ADC_mean_	1.10	24	9	10	20	1.35±0.46	0.94±0.44
	0.300.800.1400	ADC_mean_	1.10	23	10	11	19	1.31±0.44	0.94±0.44
	0.300	ADC_min_	1.30	24	8	10	21	1.70±0.62	1.04±0.49
	0.800	ADC_min_	1.00	24	5	10	24	1.43±5.24	0.86±0.41
	0.1400	ADC_min_	0.90	26	7	8	22	1.22±0.45	0.76±0.37
	0.300.800.1400	ADC_min_	0.90	26	7	8	22	1.19±4.23	0.76±0.39
Del Grande F 2016	50, 400, 800	ADC_min_	0.80	9	7	3	19	NA	NA
	50, 400, 800	ADC_mean_	1.60	9	15	3	11	NA	NA
Bonarelli C, 2014	0.600	ADC_mean_	1.65	36	5	19	5	1.74±0.48	1.58±0.47
	0.600	ADC_min_	1.28	31	10	9	15	1.51±0.59	1.10±0.26
Dodin G, 2020	0.600	ADC_min_	1.90	45	84	4	37	1.63±0.61	1.30±0.44
Hassanien OA, 2018	0.500.800	ADC_mean_	1.24	19	2	7	17	NA	NA
Romeih M, 2018	0.400.800	ADC_mean_	1.10	24	6	4	16	1.43±0.56	0.74±0.18
Teixeira PAG2016	^a^0.600	ADC_min_	1.19	16	16	14	30	NA	NA
	^b^0.600	ADC_min_	1.68	29	32	1	14	NA	NA
Song Y, 2017	0.400.800	ADC_mean_	1.13	28	7	3	13	1.60±0.48	1.24±0.46
	0.400.800	ADC_min_	0.63	27	4	8	12	0.97±0.41	0.94±0.35
Razek A, 2010	0.500.1000	ADC_mean_	1.34	21	2	1	13	1.54±0.03	1.02±0.30
Lee JH, 2020	^c^0.400.800	ADC_mean_	1.36	58	8	9	20	1.62±0.50	1.13±0.47
	^d^0.400.800	ADC_mean_	0.91	20	1	1	19	1.38±0.40	0.94±0.23
Robba T, 2017	0.800	ADC_mean_	1.45	29	5	3	7	NA	NA
Choi YJ, 2018	0, 50, 500, 800, 1400	ADC_min_	0.94	54	19	11	52	1.44±0.46	0.90±0.40
	0, 50, 500, 800, 1400	ADC_mean_	1.18	57	16	9	54	1.13±0.42	0.77±0.36
Jeon JY,2016	^e^0.400.800	ADC_mean_	1.09	19	9	6	26	1.39±0.51	0.87±0.36
	^f^0.400.800	ADC_mean_	1.49	25	17	0	18	1.56±0.61	1.01±0.33

^ab^: Result from different cut off value; ^cd^: result from all STTs and non-myxoid STTs respectively; ^e f^: result from different ROI placement.

**Table 4:** Results of Meta-regression and Subgroup Analyses for benign and malignant soft tissue tumors

Modality and group	No. study	Sensitivity (%)	Specificity (%)	PLR	NLR	Diagnostic OR	*I^2^* (%)	AUC	Q* index	^a^ *P*
Overall	27	0.80 (0.77-0.82)	0.63 (0.60-0.67)	2.37 (2.15-2.62)	0.30 (0.26-0.34)	8.61 (6.93-10.72)	42.3	0.806	0.741	0.01
**ADC measurements**										0.399
ADC_min_	12	0.79 (0.76-0.83)	0.60 (0.56-0.64)	2.21 (1.95-2.50)	0.31 (0.26-0.38)	8.23 (6.08-11.14)	31.7	0.805	0.74	
ADC_mean_	15	0.80 (0.76-0.83)	0.68(0.63-0.73)	2.61 (2.21-3.08)	0.28 (0.23-0.35)	9.08 (6.06-11.47)	56.6	0.808	0.756	
**Number of b values**										0.409
b=2	12	0.76 (0.71-0.80)	0.54 (0.49-0.59)	1.86 (1.64-2.11)	0.39 (0.32-0.47)	5.54 (4.06-7.58)	0	0.755	0.698	
b>2	15	0.83 (0.80-0.86)	0.72 (0.67-0.76)	2.91 (2.28-3.72)	0.26 (0.20-0.34)	13.06 (9.58-17.81)	38.8	0.842	0.774	
**Maximal b value**										0.858
b≤600	7	0.76 (0.70-0.81)	0.46 (0.40-0.52)	1.58 (1.39-1.81)	0.43 (0.33-0.55)	4.40 (2.95-6.57)	0	0.705	0.657	
600<b≤800	15	0.83 (0.80-0.86)	0.72 (0.68-0.76)	3.02 (2.58-3.53)	0.24 (0.19-0.29)	13.26 (9.71-18.12)	13.3	0.847	0.778	
b>800	5	0.76 (0.69-0.82)	0.73 (0.65-0.81)	2.81 (2.11-3.85)	0.33 (0.25-0.45)	8.01 (4.91-13.05)	47	0.767	0.706	
**Pathology of tumor**										0.515
All STTs	22	0.79 (0.76-0.81)	0.62 (0.59-0.65)	2.25 (2.03-2.49)	0.32 (0.28-0.37)	7.80 (6.20-9.79)	30.9	0.794	0.731	
no-myxoid STTs	5	0.88 (0.80-0.93)	0.80 (0.69-0.89)	4.34 (2.73-6.88)	0.14 (0.08-0.24)	26.69 (12.11-60.06)	58.4	0.943	0.882	
**Percentage of myxoid MTTs**										0.041
>10%	15	0.76 (0.72-0.79)	0.66 (0.61-0.70)	2.24 (1.94-2.59)	0.37 (0.31-0.44)	6.44 (4.79-8.34)	18.2	0.769	0.711	
≤10%	12	0.86 (0.82-0.89)	0.61 (0.57-0.66)	2.52 (2.19-2.89)	0.22 (0.17-0.28)	12.91 (8.96-18.61)	46.5	0.867	0.798	
**Magnet strength**										0.019
1.5T	8	0.80 (0.75-0.84)	0.49 (0.43-0.53)	1.78 (1.55-2.04)	0.33 (0.25-0.43)	6.21 (4.15-9.29)	58.6	0.790	0.728	
3.0T	19	0.80 (0.76-0.83)	0.71 (0.67-0.75)	2.83 (2.46-3.25)	0.29 (0.24-0.34)	9.96 (7.69-12.90)	24.2	0.812	0.746	
**Study design**										0.000
Retro	18	0.81 (0.78-0.84)	0.73 (0.69-0.76)	2.96 (2.56-3.41)	0.27 (0.23-0.32)	11.14 (8.58-14.47)	25.6	0.814	0.748	
Pros	9	0.77 (0.72-0.82)	0.48 (0.43-0.53)	1.66 (1.45-1.88)	0.39 (0.30-0.50)	4.98 (3.35-7.39)	38.6	0.749	0.693	
**ROI placement**										0.018
Manually over solid portion	22	0.79 (0.76-0.82)	0.63 (0.60-0.67)	2.37 (2.12-2.64)	0.30 (0.26-0.35)	8.33 (6.62-10.50)	45.1	0.801	0.737	
other	5	0.83 (0.75-0.90)	0.63 (0.54-0.72)	2.43 (1.87-3.16)	0.25 (0.16-0.39)	11.08 (5.54-22.15)	39.6	0.833	0.765	
**Total number of patients**										0.266
≤60	10	0.85 (0.80-0.89)	0.67 (0.61-0.73)	2.70 (2.19-3.32)	0.30 (0.26-0.35)	12.65 (7.93-20.19)	18.1	0.840	0.772	
>60	17	0.77 (0.75-0.81)	0.62 (0.58-0.68)	2.27 (2.03-2.55)	0.32 (0.28-0.38)	7.66 (5.97-9.82)	49.2	0.794	0.73	

^a^ Represents the *P* value of meta-regression analysis, *P* <0.05 indicates significant contribution to heterogeneity. MTTs: malignant soft tissue tumors; All STTs: all soft tissue tumors; non-myxoid STTS: non-myxoid soft tissue tumors; ADC_min_: minimum ADC value; ADC_mean_: mean ADC value; PLR: positive likelihood ratio; NLR: negative likelihood ratio; AUC, area under SROC curve.

**Table 5:** Diagnostic results of IVIM for benign and malignant soft tissue tumors

Author year	b-values (s/mm^2^)	IVIM parameters	Cutoff values (×10^-3^mm^2^/s)	TP	FP	FN	TN	Benign tumors (×10^-3^mm^2^/s)	Malignant tumors (×10^-3^mm^2^/s)
Wu H, 2017	0, 10, 20, 30, 50, 100, 200, 300, 500, 800	ADC	10.24	10	5	4	39	1.62±0.06	1.28±0.08
		D	10.13	11	10	3	34	1.35±0.05	1.06±0.06
		D*	1190	12	11	2	23	124.96±3.96	144.40±6.65
		f	90.85	4	3	10	41	14.70±0.81	14.44±1.42
Teixeira, PAG2019	a20, 40, 60, 80, 100, 200, 300, 500, 700, 900	ADC	125	18	5	11	32	1.63±0.05	1.23±0.05
		D	96	15	3	14	32	1.54±0.05	1.12±0.05
		D*	992	18	7	11	28	84.2±76.2	132.1±103.1
		f	85	16	18	13	17	0.1±0.1	0.1±0.1
	b20, 40, 60, 80, 100, 200, 300, 500, 700, 900	ADC	125	18	5	4	25	1.51±0.04	1.94±0.04
		D	99	16	3	6	27	1.42±0.04	0.94±0.05
		D*	750	18	9	4	21	90.6±78.6	153.2±108.4
		f	90	11	12	11	18	0.1±0.06	0.1±0.04
Lee SK, 2020	a0, 25, 50, 75, 100, 200, 300, 500, 800	ADC	1.31	23	8	8	22	1.47±0.35	1.17±0.49
		D	1.19	22	5	9	25	1.41±0.37	1.13±0.50
		D*	274	18	13	13	17	297±96	258±83
		f	82	17	13	14	17	1.01±0.58	0.83±0.36
	b0, 25, 50, 75, 100, 200, 300, 500, 800	ADC	1.26	20	5	3	14	1.37±0.24	0.98±0.30
		D	1.18	20	4	3	15	1.29±0.25	0.94±0.29
		D*	279	15	8	8	11	312±114	266±92
		f	88	14	6	9	13	114±61	88±36
Zhang XL 2018	0,10,20,30,40,50,75,100,150,200,400,800,1000,1500	ADC	1.33	8	7	0	11	1.90±0.43	1.27±0.38
		D	1.42	14	1	5	6	1.71±0.45	1.04±0.35

^a.b^ Results from all STTs and non-myxoid STTs respectively.

## Abbreviations

MRI: Magnetic resonance imaging
DWI: diffusion-weighted imaging
IVIM: intravoxel incoherent motion
DKI: diffusion kurtosis imaging
STTs: soft tissue tumors
ADC: apparent diffusion coefficient
MK: mean kurtosis
D*: pseudo diffusion coefficient
f: perfusion fraction
SEN: sensitivity
SPE: specificity
SROC: summary receiver operating characteristic
AUC: area under the curve
CI: confidence interval
DOR: diagnostic odds ratio
FN: false-negative
FP: false-positive
TN: true-negative
TP: true-positive
NLR: negative likelihood ratio
PLR: positive likelihood ratio
QUADAS: Quality Assessment of Diagnostic Accuracy Studies
